# Identification of nine mutant genes and establishment of three prediction models of organ tropism metastases of non‐small cell lung cancer

**DOI:** 10.1002/cam4.5233

**Published:** 2022-09-26

**Authors:** Shuchen Chen, Wanyi Huang, Zhenzhen Liu, Meizi Jin, Jielin Li, Lihui Meng, Ting Li, Yuzhu Diao, Hong Gao, Chengyu Hong, Jian Zheng, Fei Li, Yue Zhang, Dan Bi, Lin Teng, Xiaoling Li

**Affiliations:** ^1^ Department of Thoracic Medicine, Cancer Hospital of China Medical University Liaoning Cancer Hospital and Institute Shenyang China; ^2^ School and Hospital of Stomatology China Medical University, Liaoning Provincial Key Laboratory of Oral Diseases Shenyang China; ^3^ Department of Aging Science and Pharmacology, Faculty of Dental Science Kyushu University Fukuoka Japan; ^4^ Hangzhou Jichenjunchuang Medical Laboratory Co. Ltd. Hangzhou China

**Keywords:** gene mutations, NGS, NSCLC, organ tropism metastases, prediction model, targeted therapy

## Abstract

**Background:**

Most Non‐small cell lung cancer (NSCLC) patients tend to have metastases at the initial diagnosis. However, limited knowledge has been established regarding which factors, are associated with its metastases. This study aims to identify more biomarkers associated with its organ tropism metastasis and to establish models for prediction of its metastatic organs.

**Methods:**

We performed targeted next‐generation sequencing (NGS) to detect genes related to lung cancer in 272 patients with primary advanced NSCLC from Northeast China. We adopted Fisher test, multivariate logistic regression analysis to identify metastasis‐related gene mutations and to establish prediction models.

**Results:**

Mutations of *EGFR* (*p* = 0.0003, OR = 2.554) (especially *EGFR* L858R [*p* = 0.02, OR = 2.009]), *ATM* (*p* = 0.008, OR = 11.032), and *JAK2* (*p* = 0.009, OR = Inf) were positively and of *TP53* exon4mut (*p* = 0.001, OR = 0.173) was negatively correlated with lung metastasis, and those of *CSF1R* (*p* = 0.01, OR = Inf), *KIT* (*p* = 0.03, OR = 4.746), *MYC* (*p* = 0.05, OR = 7.938), and *ERBB2* (*p* = 0.02, OR = 2.666) were positively correlated with pleural dissemination; those of *TP53* (*p* = 0.01, OR = 0.417) was negatively, while of *SMAD4* (*p* = 0.03, OR = 4.957) was positively correlated with brain metastasis of NSCLC. Additionally, smoking history (*p* = 0.004, OR = 0.004) was negatively correlated with pleural dissemination of NSCLC. Furthermore, models for prediction of lung metastasis (AUC = 0.706), pleural dissemination (AUC = 0.651), and brane metastasis (AUC = 0.629) were established.

**Conclusion:**

Taken together, this study revealed nine mutant genes and smoking history associated with organ tropism metastases of NSCLC and provided three models for the prediction of metastatic organs. This study enables us to predict the organs to which non‐small cell lung cancer metastasizes before it does develop.

## Graphical abstract

This study identified ten factors including nine gene mutations and smoking history as predictors for the organ tropism metastasis of NSCLC through analyses of NGS data and other clinical factors. Then three models were established to predict lung metastasis, brain metastasis, and pleura dissemination of NSCLC using these factors.

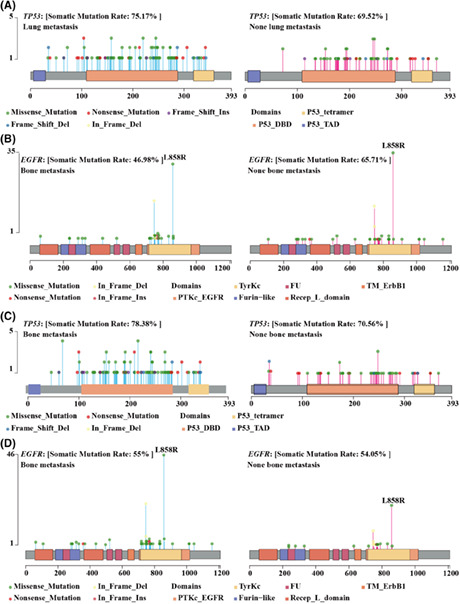

## 1 INTRODUCTION

Lung cancer is a malignant tumor with the second‐highest incidence and the highest mortality in the world.^1^ For all stages, Only 10% to 20% of lung cancer patients survive over 5 years after diagnosis in most countries, due to its fast progression and metastatic potency.^2^ Metastases have been estimated to account for approximately 90% of all lung cancer caused deaths.^3^ The preference metastatic organs of lung cancer include brain, pleural, bone, liver, adrenals and lung.^4^ Patients with metastases usually have shorter median overall survival and lower quality of life. The prevention and early diagnosis of metastases can help prolong the survival and improve the life quality of patients.^5^ Therefore, it is of great significance to study the molecular mechanisms of and correlated risk factors of metastases. Previous studies have revealed that clinical factors such as age, gender, primary sites of tumors, smoking history, and pathology are associated with metastases of lung cancer.^6^, ^7^, ^8^, ^9^ A series of gene mutations and their related pathways associated with metastases also have been identified. For example, Wingless and INT‐1 (WNT) and mitogen‐activated protein kinases (MAPK) signaling pathways and alterations of *EGFR*, *anaplastic lymphoma kinase* (*ALK*), *Kirsten rat sarcoma viral oncogene homolog* (*KRAS*), *matrix metalloproteinase* (*MMP*), *mesenchymal‐epithelial transition* (*MET*) and *Live kinase B1* (*LKB1*) genes have been identified to be involved in brain metastasis9, and WNT, MAPK and NF‐kappaB (NFκB) signaling pathways in bone metastasis and *EGFR* mutations in lung metastasis of lung cancer.^10^ However, knowledge of mutated genes and molecular mechanisms associated with lung cancer metastases is still limited, so further studies are needed to identify more key genes.

Clinical next‐generation sequencing (NGS) assays include whole genome sequencing (WGS), whole exome sequencing (WES) and targeted next‐generation sequencing. With the wide application of clinical NGS in detection of cancer patients, unprecedented scale of information about tumor‐related genomic alterations are being accumulated, making it easier to identify more gene mutations associated with lung cancer metastasis.^11^

Non‐small cell lung cancer (NSCLC) is the most common form of lung cancer with more than a half of patients have distant metastases at the time of diagnosis.^12^ The incidence of ipsilateral lung metastasis is estimated over 60%, of contralateral lung metastasis 26%–28%, of bone metastasis 35%–43%, of liver 18%–20%, and of adrenal metastasis 21%–27%.^13^ In this study, we identified nine mutant genes and a clinical factor that showed significant correlation with brain, lung, and pleural metastases of NSCLC and established three models for prediction of these metastases, respectively.

## 2 MATERIALS AND METHODS

### 2.1 Patients

This retrospective cohort consisted of 272 patients diagnosed of advanced non‐small cell lung cancer who were admitted to the Department of oncology, Liaoning Cancer Hospital and Institute from 2016 to 2020. The essential information including age, smoking history and gender of all patients was collected. Then the pathological types, primary sites, and metastatic sites of the patients were detected by imaging and pathological examination. The genotypes of 63 lung cancer associated genes in tumor tissues of the primary sites were determined using targeted NGS method after diagnosis.

This study was carried out with ethics committee approval by the Medical Ethics Committee of Liaoning Cancer Hospital and Institute and signed informed consents were collected from all patients.

### 2.2 Targeted NGS

DNA was extracted from paraffin‐embedded tissues of the primary site using AllPrep DNA/RNA mini Kit (Qiagen 80,204) according to its instructions. The libraries of gDNA were constructed according to the manufacturer's protocols by a KAPA Hyper Prep kit (Kapa Biosystems). A panel of 63 lung cancer‐related genes was used to enrich the gDNA libraries. The custom‐designed capture probes of the panel were manufactured by Agilent, USA. The enriched gDNA libraries were amplified with P5/P7 primers and qualified by the 2200 Bioanalyzer (Agilent Technologies) and quantified by the qBittorrent (version 3), followed by being sequenced on the Hiseq X10 platform (Illumina). The NGS data were primary analyzed using trimmomatic‐0.36. Sequence reads were aligned against human reference genome (version GRCh37/hg^14^) using bwa (version 0.7.10). Samtools (version 1.3.1) and pindel (version 0.2.5b8, 20,151,210) were used to identify candidate somatic mutations in the targeted regions. Finally, filter alignment and sequencing artifacts were conducted using IGV (Integrative Genomics Viewer).

### 2.3 Statistical analyses

The Fisher tests, multiple logistic regressions were performed using fisher. test and glm functions of R version 4.0.4, respectively. *p‐* value <0.05 indicates statistically significant and < 0.01 statistically very significant. Odds ratio (OR) > 1 means higher risk of occurrences of metastases and < 1 means lower risk. The logistic regression model models and receiver operating characteristic (ROC) curves were constructed by pROC of R version 4.0.4 using our own data and published data of other studies.^15^, ^16^, ^17^, ^18^, ^19^ Threshold indicates the probability of occurrences of metastasis of each sample and each point on the ROC curve corresponds to a threshold. The sensitivity and specificity of the model were established using an optimized threshold value which was determined by Youden's index.

## 3 RESULTS

### 3.1 Characteristics of patients

The age range of the 272 enrolled NSCLC patients was 28–97 with the median age of 63. There are 143 (52.57%) male and 132 (47.43%) female. One hundred and eight (39.71%) patients had a history of smoking and 159 (58.46%) did not. Histopathological examination showed that the tumors of 210 (77.21%) patients were adenocarcinoma, of 28 (10.29%) squamous cell and of 34 (12.5%) other types of NSCLC. The proportion of squamous cell carcinoma patients is lower than normal in this cohort because their lack of targeting gene mutations of drugs, which may make the statistical analysis results based on pathological types be unreliable. Primary sites of the tumor of 104 (38.24%) patients were in the right lung and of 164 (60.29%) in the left lobes. Metastases had been developed in most of the patients at the initial diagnosis. The tumors of 227 (83.46%) patients were in stage IV and of 45 (16.54%) in stage III. Lung metastasis was detected in 109 (40.07%), pleural dissemination (pleural effusion, pericardial effusion and nodes in pleura) in 93 (34.19%), brain metastasis in 48 (17.65%), bone metastasis in 78 (28.68%), and liver metastasis in 25 (9.19%) patients (Table 1).

**TABLE 1:** Baseline and clinicopathological information of 272 NSCLC patients

Patient characteristics	Subgroup	Statistics
All patients		272 (100%)
Median age (range)		63 (28–97)
Sex	Male	143 (52.57%)
	Female	129 (47.43%)
Primary tumor site	Right lung	157 (57.72%)
	Left lung	104 (38.24%)
Stage	III	45 (16.54%)
	IV	227 (83.46%)
Histology	Adenocarcinoma	217 (77.21%)
	Squamous cell	30 (10.29%)
	NSCLC other	36 (12.50%)
Smoking history	Yes	108 (39.71%)
	No	160 (58.82%)
	Unknown	4 (1.47%)
Metastatic site	Lung	109 (38.52%)
	Brain	48 (16.96%)
	Bone	78 (27.56%)
	Liver	25 (8.83%)
	Pleural effusion	93 (32.86%)

### 3.2 Gene alterations of patients

**FIGURE 1:**
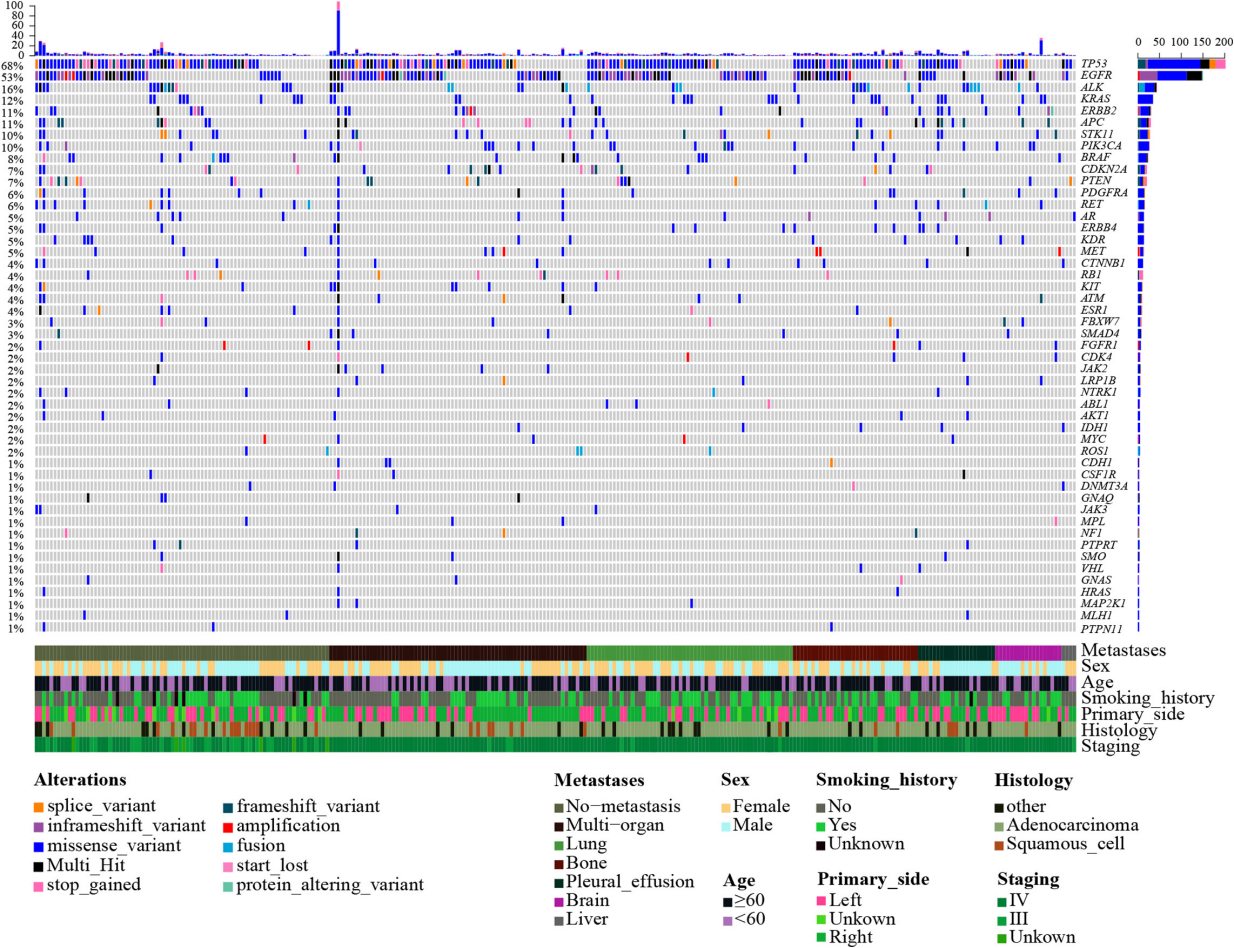
Mutational landscape of 272 NSCLC patients. Each column represents an individual patient. Each row represents a gene with their different mutation types were indicated by different color. Names of the genes were indicated on the right side. The frequencies of gene mutations were indicated on the left side and the numbers of mutated genes of each individual were indicated on the top. Corresponding clinicopathological and baseline information of each patient is marked at the bottom of the figure.

To investigate the genomic alterations of these patients, we adopted targeted NGS to detect the variations of 63 genes associated with the development and therapies of lung cancer at the time of initial diagnosis. Gene alterations such as missense mutations, early termination (stop gained), gene fusions, copy number alterations (CNA)/amplifications and other types of mutations were detected in 264 (97.06%) of 272 patients. Alterations of *TP53* were detected in 186 (68.38%) patients, of *EGFR* in 145 (52.57% mutations and 10.66% amplifications) patients, of *ALK* in 41 (15.07%; 5.15% fusions and 11.40% mutations) patients, of *KRAS* in 33 (12.13%) patients and of *ERBB2* (*HER2*) in 31 (11.40%; 11.03% mutations and 1.47% amplifications) (Figure 1). In addition, the mutation frequencies of *anaphase‐promoting complex* (*APC*), *STK11* and *PIK3CA* were also higher than 10% in this cohort (Figure 1). We further analyzed the mutation sites of *TP53* and *EGFR* (Figure S1). Mutations of *TP53* were harbored in exons 3–10, and predominantly clustered in the DNA‐binding domain including exons 4–8 with the mutations of 24 patients harbored in exon 4 (*TP53* exon 4 mut), 55 in exon 5 (*TP53* exon 5 mut), 36 in exon 6 (*TP53* exon 6 mut), 50 in exon 7 (*TP53* exon 7 mut), and 34 in exon 8 (*TP53* exon 8 mut). For *EGFR*, most mutations were harbored in exons 19 and 21. An amino acid substitution (Leucine to Arginine) in exon 21 (*EGFR* L858R) was detected in 60 patients and in‐frame deletions in exon 19 (*EGFR* exon 19 del) were detected in 40 patients (Figure S1).

### 3.3 Variables associated with organ tropism metastases of NSCLC in this cohort

More than 70% of the lung cancer patients developed distant or lung metastasis and frequencies of mutant genes were different among groups with different organ tropism metastases. To explore variables associated with organ tropism metastases of NSCLC, we performed univariate analysis between baseline factors and metastatic organs of NSCLC using Fisher tests. Our results showed that adenocarcinoma account for a larger proportion in lung metastasis patients than no lung metastasis ones (94.68% vs. 84.03%, *p*‐value = 0.01, OR = 3.368) (Table 2), suggesting that pathological types of NSCLC is associated with lung metastasis but this may be inaccurate considering the lower proportion of squamous cell carcinoma patients of this cohort. In addition, we found that smoking history (27.78% vs. 46.63%, *p* = 0.004, OR = 0.442) (Table 2) was negatively correlated with pleural dissemination.

**TABLE 2:** Fisher test of the correlative risk factors with metastases of tumor to different organs

Factors	Metastatic organ		OR (95% CI)	*p*‐value
	Lung metastasis	No lung metastasis		
*EGFR* **	73 (66.97%)	72 (44.17%)	2.554 (1.503, 4.392)	0.0003
*EGFR* L858R*	35 (32.11%)	31 (19.02%)	2.009 (1.105. 3.670)	0.02
*EGFR* Exon19Del	21 (19.27%)	25 (15.34%)	1.316 (0.657, 2.616)	0.413
*TP53* Exon4mut**	3 (2.75%)	23 (14.11%)	0.173 (0.032, 0.596)	0.001
*JAK2* **	5 (4.59%)	0	Inf (1.400, Inf)	0.009
*ATM* **	7 (6.42%)	1 (0.61%)	11.032 (1.385, 502.818)	0.008
Adenocarcinoma**	89 (94.68%)	121 (84.03%)	3.368 (1.191, 11.784)	0.01
	Pleural dissemination	No pleural dissemination		
*CSF1R* **	4 (4.30%)	0	Inf (1.293, Inf)	0.01
*ERBB2* *	15 (16.13%)	12 (6.70%)	2.666 (1.106, 6.561)	0.02
*KIT* *	7 (7.53%)	3 (1.68%)	4.746 (1.052, 29.148)	0.03
*MYC* **	4 (4.30%)	1 (0.56%)	7.938 (0.771, 395.279)	0.05
Smoking history**	25 (27.78%)	83 (46.63%)	0.442 (0.243, 0.784)	0.004
	Brain metastasis	No brain metastasis		
*TP53* **	25 (52.08%)	162 (72.32%)	0.417 (0.210, 0.832)	0.009
*TP53* Exon4mut	1 (2.08%)	25 (11.16%)	0.17 (0.004, 1.094)	0.06
*TP53* Exon5mut	4 (8.33%)	45 (20.09%)	0.363 (0.090, 1.075)	0.06
*SMAD4* *	4 (8.33%)	4 (1.79%)	4.957 (0.888, 27.671)	0.03
	Bone metastasis	No bone metastasis		
*TP53* Exon6mut*	4 (5.13%)	30 (15.46%)	0.297 (0.073, 0.886)	0.02

*Note*: Red asterisk ‘*’, indicates positive correlation; double red asterisk ‘**’, significant positive correlation; blue asterisk ‘*’, negative correlation; double blue asterisk ‘**’, negative correlation significant. OR is short for odds ratio and CI is short for confidence interval.

**FIGURE 2:**
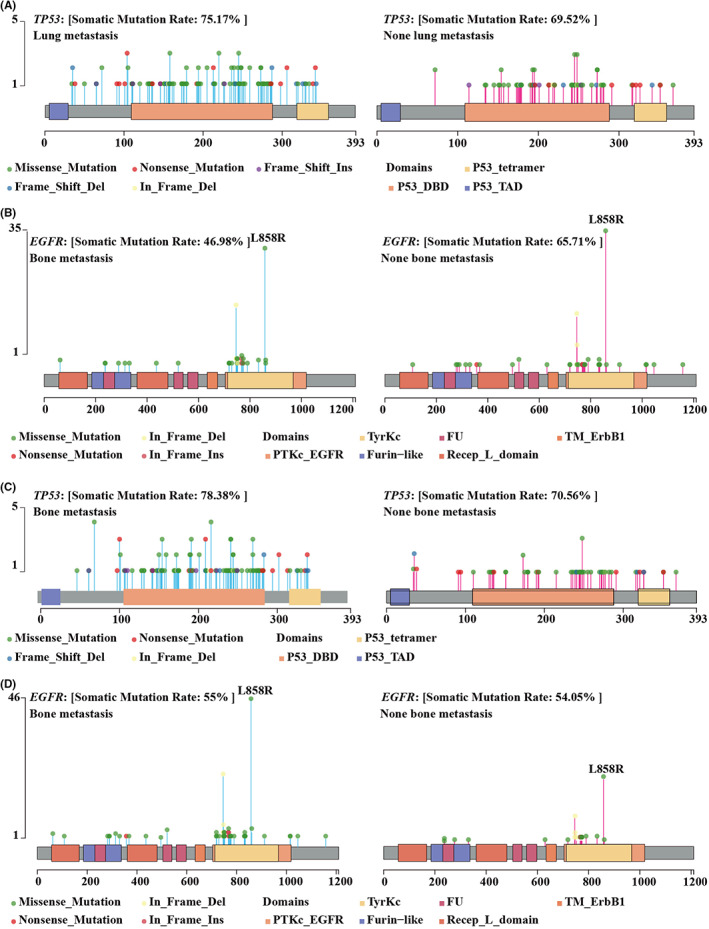
Mutation sites of *TP53* and *EGFR* of subgroups with different organ metastases. (A) Mutation sites of *TP53* of none lung metastasis and lung metastasis patients. (B) Mutation sites of *EGFR* of none lung metastasis and lung metastasis patients. (C) Mutation sites of *TP53* of bone none metastasis and lung metastasis patients. (D) Mutation sites of *EGFR* of none lung metastasis and bone metastasis patients. Left, gene mutation sites of none lung/bone metastasis patients; Right, gene mutation sites of lung/bone metastasis patients. Horizontal axes indicate numbers of protein sites; vertical axes indicate number of mutations. The hight of each straight line indicates mutation number of the corresponding sites of subgroup. Ins is short for insertion; Del, deletion; DBD, DNA binding domain; TAD, transactivtion domain; PTK, protein tyrosine kinase; TyrK, tyrosine kinase; TM, transmembrane.

We further performed univariate analysis between gene mutations and metastatic organs of NSCLC to identify molecular markers of organ tropism metastases. The results showed that the mutation frequencies of *EGFR* (66.97% vs. 44.17%, *p*‐value = 0.0003, OR = 2.554), *ATM* (6.42% vs. 0.61%, *p*‐value = 0.0078, OR = 11.032) and *JAK2* (4.59% vs. 0%, *p*‐value = 0.009, OR = Inf) in lung metastasis patients were higher in lung metastasis patients than in no‐lung metastasis ones (Table 2), indicating that mutations of *EGFR*, *ATM*, and *JAK2* were positively and significantly (Figure 2A,B). Lung cancer is easy to spread to the pleura. We found that mutations of *CSFIR* (4.30% vs. 0%, *p*‐value = 0.01, OR = Inf), *MYC* (4.30% vs. 0.56%, *p*‐value = 0.05, OR = 7.938), *KIT* (7.53% vs. 1.68%, *p*‐value = 0.03, OR = 4.746) and *ERBB2* (16.13% vs. 6.70%, *p*‐value = 0.02, OR = 2.666) were positively correlated with pleural dissemination (Table 2). Furthermore, mutations of *SMAD4* (8.33% vs. 1.79%, *p*‐value = 0.03, OR = 4.954) was positively correlated with brain metastasis while mutant *TP53* (52.08% vs. 72.32%, *p*‐value = 0.009, OR = 0.417) was negatively (Table 2).

To explore the effect of mutations harbored in different sites of *TP53* and *EGFR* on metastases of NSCLC, we performed univariate analysis and marked the mutation site of the two genes of metastasis and none metastasis subgroups. We found that *EGFR* L858R (32.11% vs. 19.02%, *p*‐value = 0.02, OR = 2.009) was positively while *TP53* exon4mut (2.75% vs. 14.11%, *p*‐value = 0.001, OR = 0.173) was negatively correlated with lung metastasis (Table 2, Figure 2), which is consistent with previous studies.^13^ Besides, *TP53* exon6mut was negatively correlate to bone metastasis (52.08% vs. 72.32%, *p*‐value = 0.009, OR = 0.417) (Figure 2A,D). However, *TP53* mutations except for *TP53* exon6mut (*TP53* exc. exon6mut) (69.23% vs. 51.03%, *p*‐value = 0.007, OR = 2.153) was positively correlated to bone metastasis very significantly (Table 2, Figure 2).

### 3.4 Prediction models for organ tropism metastases of NSCLC

To further determine the association of the factors identified by univariate analyses with NSCLC metastases, all of them were then included in logistic multivariate analyses. The results confirmed that mutations of *EGFR* (*p* = 0.0008, OR = 2.584) and of *ATM* (*p* = 0.04, OR = 12.598) were positively correlated with while, *TP53* exon4mut (*p* = 0.004, OR = 0.154) and *RET* (*p* = 0.07, OR = 0.109) was negatively correlated with lung metastasis. Mutations *ALK* was positively correlated with lung metastasis but was not statistically significant (*p* = 0.08). Additionally, we determined that mutations of *KIT* (*p* = 0.02, OR = 6.076) and *TP53* exon4mut (*p* = 0.01, OR = 2.956) were positively and smoking history (*p* = 0.008, OR = 0.416) was negatively correlated with pleural dissemination. Furthermore, *TP53* (*p* = 0.02, OR = 5.667) was negatively correlated with brain metastasis significantly while *SMAD4* (*p* = 0.02, OR = 6.076) was positively correlated with significantly (Table 3).

**TABLE 3:** The multivariate logistic regression analysis for lung metastasis, pleural dissemination and brane metastasis

Variable	OR (95% CI)	*p*‐value
Lung metastasis		
(Intercept)	0.459 (0.217, 0.952)	0.04
Pathology	1.028 (0.680, 1.542)	0.89
*EGFR*	2.584 (1.491, 4.545)	0.0008
*TP53* exon4mut	0.154 (0.035, 0.477)	0.004
*JAK2*	NA	0.98
*RET*	0.109 (0.0039, 0.757)	0.07
*ATM*	12.598 (1.583, 302.817)	0.04
*ALK*	0.529 (0.218, 1.191)	0.14
Pleural dissemination		
(Intercept)	0.552 (0.390, 0.774)	0.0007
Smoking	0.418 (0.232, 0.734)	0.003
*CSF1R*	NA	0.99
*TP53* exon4mut	2.956 (1.260, 7.100)	0.01
*KIT*	6.076 (1.499, 30.478)	0.02
*MYC*	6.991 (0.846, 145.817)	0.1
Brain metastasis		
(Intercept)	0.342 (0.207, 0.546)	1.00E‐05
*TP53*	0.416 (0.217, 0.796)	0.008
*SMAD4*	5.667 (1.261, 25.550)	0.02

**FIGURE 3:**
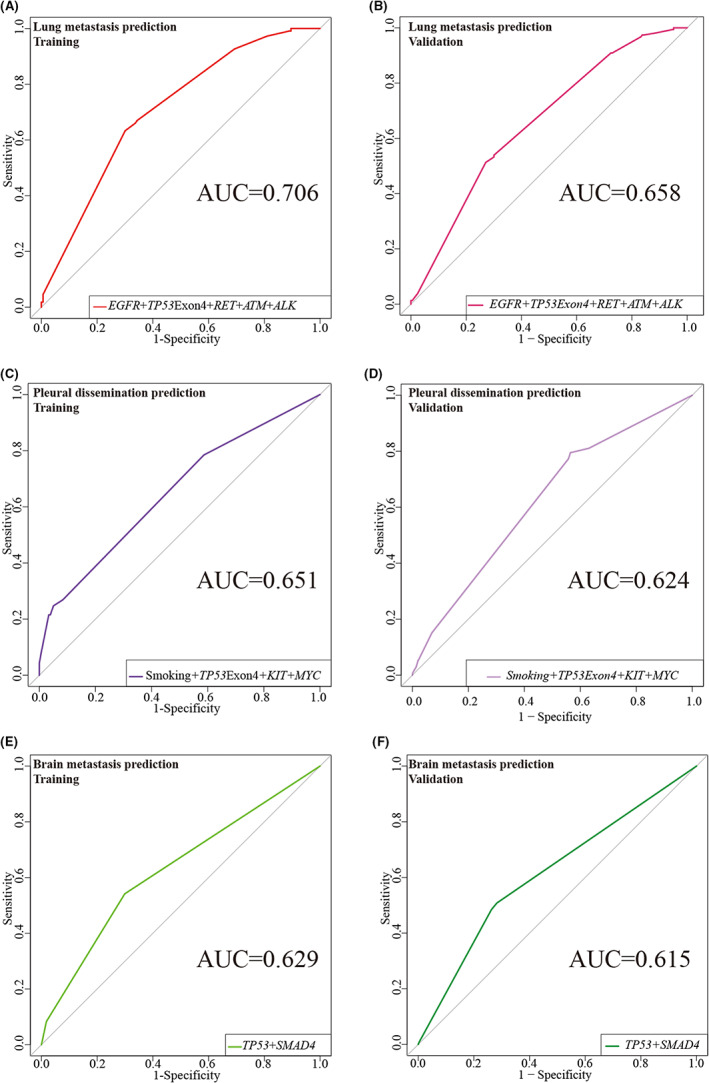
ROCs of the multivariable logistic regression models for prediction of lung metastasis, pleural dissemination and brane metastasis. (A, B) ROCs of the multivariable logistic regression model of training and validation groups for prediction of lung metastasis. (C, D) ROC of the multivariable logistic regression model of training and validation groups for prediction of pleural dissemination. (E, F**)** ROC of the multivariable logistic regression model of training and validation groups for prediction of brane metastasis.

Based on logistic multivariate analyses significant variates were combined to construct 3 logistic models for prediction of lung pleural and brane metastasis, respectively. The AUC (area under the curve) of the lung metastasis prediction model was 0.706, of pleural dissemination 0.651 and of brane metastasis 0.629 (Figure 3, Table 4). Then we selected the cutoff threshold according to the highest Youden index. The cutoff threshold of the lung metastasis prediction model is 0.484 with a sensitivity of 63.30% and a specificity of 69.94% (Table 4). The threshold of the pleural dissemination and brain metastasis prediction models are 0.277 (sensitivity of 78.49%, specificity of 42.34%) and 0.19 (54.17%, 70.09%) (Figure 3, Table 4), respectively.

**TABLE 4:** Sensitivity and specificity at the respective cutoff threshold of models

Prediction models	AUC (95% CI)	Threshold	Sensitivity	Specificity
Lung metastasis (*EGFR* + *TP53* exon4mut + *RET* + *ATM* + *ALK*)				
Training	0.706 (0.649, 0.764)	0.484	63.30%	69.94%
Validation	0.658(0.605, 0.711)	0.531	51.30%	72.91%
Pleural dissemination (Smoking + *TP53* exon4mut + *KIT* + *MYC*)				
Training	0.651 (0.587, 0.714)	0.277	78.49%	41.34%
Validation	0.624(0.579, 0.668)	0.131	79.5%	43.5%
Brain metastasis (*TP53* + *SMAD4*)				
Training	0.629 (0.549, 0.708)	0.19	54.17%	70.09%
Validation	0.615(0.564, 0.666)	0.27	50.79%	71.75%

*Note*: These models correspond to those in Figure 3.

## 4 DISCUSSION

Most NSCLC patients are at advanced stages and have developed metastases at the initial diagnosis. It was hypothesized that metastases of cancers involved seven steps which correlated with cancer‐cell surface proteins, cytokines, chemokines and growth factors in the tumor‐micro‐environment19. Additionally, they proposed that CXCL12/CXCR4 signaling pathways, *EIF4EBP1*, *EGFR*, *ERBB2* and *VEGFR2* involved in lung cancer brain metastasis, L1CAM‐mediated ERK1/2 signaling, *EGFR* and *KRAS* involved in lung cancer bone metastasis of and *type‐1 insulin‐like growth factor receptor* (*IGF‐1R*) involved in lung cancer liver metastasis19. In this study we detected the somatic mutations of 272 NSCLC patients (stage III or IV) using targeted NGS. Although the frequency of mutation of *ALK* (15.07% vs. 5%–10%) and *ERBB2* (11.40% vs. 2%–4%) was higher than previous reported,^20^ the frequency of *ALK* fusions (5.15% vs. 7.8%) was not.^21^ Through subsequent univariate analyses we identified variates including nine mutant genes and smoking history that significantly correlated with the organ tropism metastases of NSCLC. Then we established three models for prediction of lung metastasis (*EGFR* + *TP53* exon4mut + *RET* + *ATM + ALK*), pleural dissemination (Smoking+ *TP53* exon4mut + *KIT*) and brane metastasis (*TP53* + *SMAD4*), respectively. The AUC of the three prediction models are greater than 0.6. Both specificity and sensitivity of the lung metastasis prediction model are greater than 60%, sensitivity of the pleural dissemination prediction model and the specificity of brain metastasis model are greater than 70%. These characters of the models suggest that a comprehensive assessment of and gene mutations and other risk factors will be of great value in predicting organ tropism metastases of NSCLC and that examination of corresponding organs should be performed during follow‐up of patients with these variates.

Lung is a major site of metastases formation from a variety of malignancies such as breast and colon cancer, melanoma and especially primary lung cancer. In this cohort about 40% of the patients had lung metastasis. Previous studies showed that the mutations of *KRAS* and *BRAF* had positive correlations to lung metastasis of colorectal cancer and papillary thyroid carcinoma, respectively.^22^, ^23^ We found that mutations of *EGFR* were positively correlated with lung metastasis of NSCLC, which is consistent with previous studies.^24^ Additionally, *EGFR* L858R exhibited significant positive correlation with lung metastasis while *EGFR* exon19del did not, which might provide a new way to explain the poorer prognosis of *EGFR* L858R. Besides, *ATM* mutations suggested an increase while *TP53* exon4mut a decrease in the risk of lung metastasis of NSCLC. Olaparide has a significant effect on both castration resistant prostate cancer (CRPC) and ovarian cancer patients with *ATM* mutation,^25^, ^26^ and a multi‐center clinical trial called ORION has been carried out for olaparide in the treatment of advanced NSCLC. In addition, a recent study showed that a metastatic colorectal cancer patient with *ATM* loss of function mutation benefited from an ATR inhibitor M6620 (VX‐970) monotherapy.^27^ We can speculate that there will be drugs targeting *ATM* to treat NSCLC in the future.

So far, only a few mutant genes have been revealed to be associated with pleural dissemination. Guo et al. (2016) reported that some mutated genotypes of *EGFR* had positive correlation with pleural dissemination.^13^ Our results showed that mutations of *TP53* exon4mut, *KIT* and *MYC* positively correlated with the pleural dissemination of NSCLC. *KIT* is a tyrosine kinase receptor‐encoding gene with its mutations occurring in about 75%–80% gastrointestinal stromal tumors (GIST), 9.5% in melanoma, and 3% in lung cancer. Several TKIs like Imatinib, Sunitinib and Nilotinib were identified to have specificity for *KIT*.^28^ *MYC* is an oncogene that participate in regulation of 20% cancers so it is a hot topic to development drugs targeting MYC and its related pathways.^29^ However, there remains no drug directly targeting MYC at present. Pleural dissemination of lung cancer usually leads to malignant pleural effusion which could shorten survival of patients. Our findings showed that *KIT*, *MYC*, and *TP53* exon4mut mutations may suggest a high risk of pleural dissemination. Thus, applications of drugs targeting them or their associated pathways in the treatment of advanced NSCLC would prolong the survival of patients by reducing the risk of pleural invasion. Besides, we found that smoking history negatively correlated with pleural dissemination, which is consistent with previous studies^30^ but smoking should not be promoted because it can increase the risk of multiple cancers.^31^

Lung cancer is prone to brain metastasis and it is estimated that advanced lung cancer contributes to almost half of brain metastasis patients.^32^ Many factors including pathology, age, level of tumor markers (Neuron‐specific enolase (NSE) and Carcinoembryonic antigen (CEA)) and tumor‐associated gene mutations have been identified to associate with brain metastasis. Previous researches showed that activation of Ras, Wnt, and PIK3A pathways promote brain metastasis.^32^ Alterations of multiple genes such as *EGFR*, *ALK*, *LKB1*, *KRAS*, *HOXB9*, *LEF1*, *ANGPT4*, *PDGFRB*, *YAP1*, and *MMP13* have been found to associated with brain metastasis of NSCLC9. Herein, our results showed that mutations of *SMAD4* positively correlated with brain metastasis while mutations of *TP53* negatively did. SMAD4 is a transcription factor in the TGF‐β/BMP–SMAD4 signaling pathway and participates in the regulation of tissue homeostasis, embryonic development, epithelial‐to‐mesenchymal transition (EMT) and extracellular matrix remodeling. Mutation frequency of SMAD4 is estimated about 50% in pancreatic cancer, about 30% in colon cancer whereas the frequencies in prostate, breast, liver and lung cancer are lower9.^33^, ^34^ Functional *SMAD4* is a tumor suppressor and its inactivation promotes lung cancer metastasis through de‐repression of PAK3 by miRNA regulation.^35^ However, there has been no studies showing the contribution of mutation of *SMAD4* to the brain metastasis of lung cancer prior to our research. Although mutation frequency of *SMAD4* is high in pancreatic and colon cancer, there still are no drugs targeting *SMAD4*. Previous studies have shown that mutations of both *EGFR* exon 19 del and *EGFR* L858R can increase the risk of brain metastasis of NSCLC24.^36^, ^37^ We also found positive correlation between mutations of *EGFR* and the occurrence of brain metastasis but it was not statistically significant here (*p* = 0.11). To confirm this correlation, mechanism studies and larger sample size surveys need to be conducted in the future.

*TP53*, one of the most frequently mutated tumor suppressor genes in human cancers, expresses a transcription factor p53 which contains the tetramerization motif, the transactivation motif and the DNA‐binding domain.^38^, ^39^ The frequency of simultaneous mutations of *TP53* with EGFR or KRAS is higher than 5% in both Western and Asian lung adenocarcinoma patients while it is significantly lower in lung squamous cell carcinoma patients of the two major groups.^40^ Most of the somatic missense mutations occur within the DNA‐binding domain of p53 in cancers.^41^ Germline TP53 mutations account for about 70% of families with Li‐Fraumeni syndrome which is associated with hereditary of several cancers including lung adenocarcinoma.^38^, ^42^ TP53 p.R175 and p.R248 were identified as the germline mutated sites with the highest variant rates in Chinese tumor patients with Li‐Fraumeni syndrome or Li‐Fraumeni‐like syndrome38. So far no sites have been determined as a founder mutation for Chinese tumor patients while *TP53 R337H* was identified as a founder mutation in Brazilian patients with adrenocortical tumors42. In addition to initiation and progression of cancers *TP53* mutations also promote metastases by facilitating faster proliferation and evolution of tumors and mutations and expression changes of genes related to metastases39. Herein, we found that *TP53* exon4mut positively correlated with lung metastasis but negatively with pleural dissemination and *TP53* exon6mut positively correlated with bone metastasis. It is an interesting issue worthy of further study to explore why *TP53* exon4mut inhibit pleural dissemination of NSCLC.

## 5 CONCLUSION

Our study has identified a set of potential predictors and established three models of organ tropism metastases of NSCLC. Although the mechanisms of their involvement in organ tropism metastases need to be further studied, these potential biomarkers could be used as early warning signals to prevent the occurrence of metastases and would directed target therapy of NSCLC in the future.

## AUTHOR CONTRIBUTIONS

**Shuchen Chen:** Data curation (lead); formal analysis (lead); writing – original draft (lead). **Wanyi Huang:** Data curation (equal); formal analysis (equal). **Zhenzhen Liu:** Data curation (equal). **Meizi Jin:** Data curation (equal). **Jielin Li:** Data curation (equal). **Lihui Meng:** Data curation (equal). **Ting Li:** Data curation (equal). **Yuzhu Diao:** Data curation (equal). **Yuhong Gao:** Data curation (equal). **Cheng Yu:** Investigation (equal). **Jian Zheng:** Data curation (equal). **Fei Li:** Data curation (equal). **Yue Zhang:** Formal analysis (supporting); writing – original draft (supporting). **Dan Bi:** Formal analysis (supporting). **Lin Teng:** Formal analysis (supporting). **Xiaoling Li:** Data curation (lead); formal analysis (lead); funding acquisition (lead); methodology (lead); project administration (lead); writing – review and editing (lead).

## FUNDING INFORMATION

This research was supported by the funding from the Liaoning province Department of Human Resources and Social Security and Lung Cancer Precision Medicine Research Funding of Huilan Charity‐Hausen Pharmaceutical [HL‐HS2020‐28].

## CONFLICTS OF INTEREST

The authors declare that they have no competing interests.

## ETHICS APPROVAL AND CONSENT TO PARTICIPATE

Approval for the study was obtained from the Institutional Review Board/Ethics Committee of the Medical Ethics Committee of Liaoning Cancer Hospital and Institute and signed informed consents were collected from all patients.

## Supporting information


Table S1

Figure S1

## Data Availability

The datasets generated and analyzed during the current study are available from https://ngdc.cncb.ac.cn/search/?dbId=&q=+PRJCA 007674.
