# Supplementary material for: Identification of nine mutant genes and establishment of three prediction models of organ tropism metastases of non‐small cell lung cancer
Source: Cancer Med. 2022 Sep 26;12(3):3089–100. doi: 10.1002/cam4.5233 (PMC9939125; doi:10.1002/cam4.5233)
Supplement: Supplementary file 1 — Table S1 [file CAM4-12-3089-s002.docx]

**Supplementary**

**
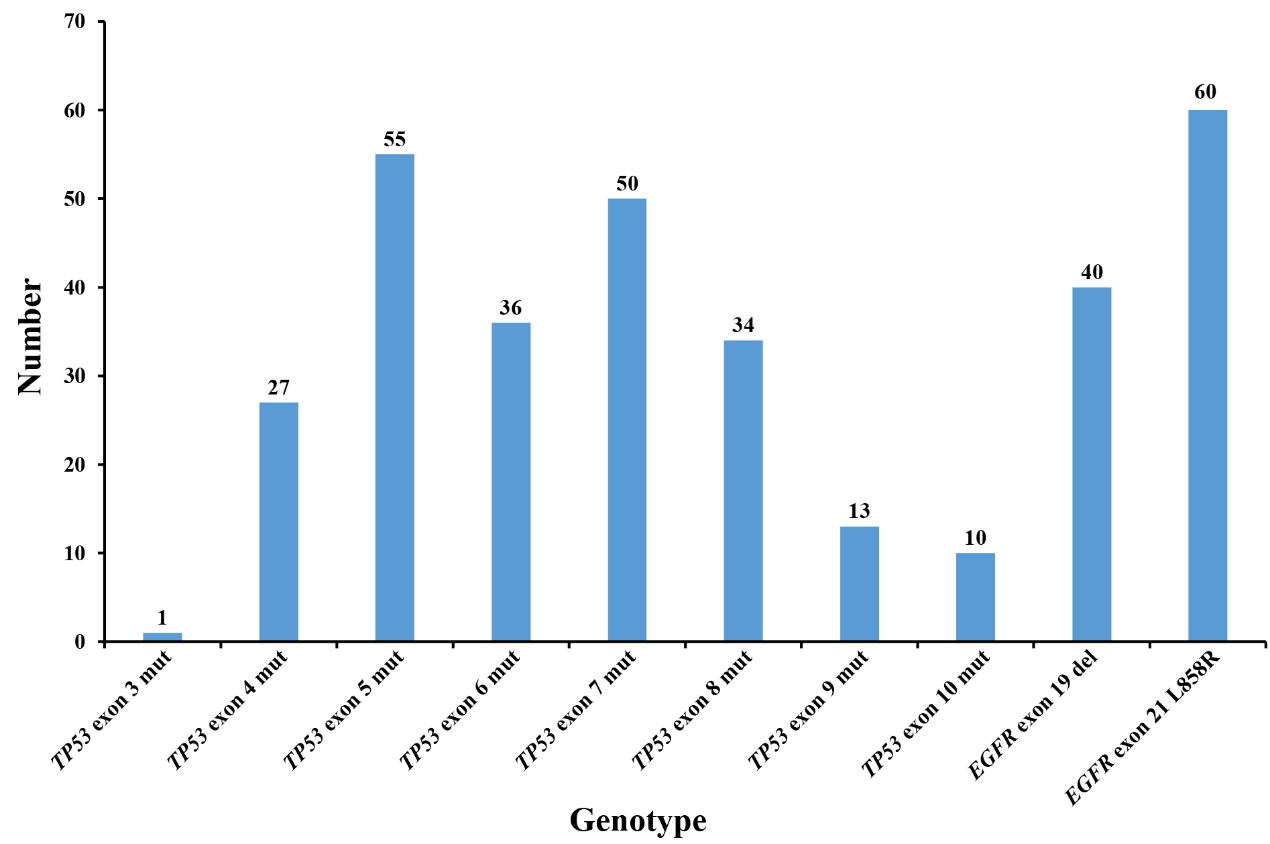
**

**Supplemental Fig. 1** The numbers of patients with different genotypes of *TP53* and *EGFR* mutations.

Genotypes of genes were indicated at the bottom of the lateral axis.

**Supplemental Table 1** Single factor Fisher test of Correlation between clinical factors and metastasis.

| **Variation** | | **Cancer metastases (%)** | | **OR (95% CI)** | **P-value** |
| --- | --- | --- | --- | --- | --- |
|  |  | Lung**^+^** (n=107) | Lung**^–^** (n=176) |  |  |
| Sex | Male | 57 (52.29%) | 72 (44.17%) | 1.384 (0.827, 2.321) | 0.216 |
|  | Female | 52 (47.71%) | 91 (55.83%) |  |  |
| Age | <60 | 40 (36.70%) | 57 (34.97%) | 1.078 (0.629, 1.84) | 0.797 |
|  | ≥60 | 69 (63.30%) | 106 (65.03%) |  |  |
| Smoking history | Yes | 44 (40.37%) | 64 (34.96%) | 1.005 (0.592, 1.7) | 1 |
|  | No | 65 (59.63%) | 95 (65.03%) |  |  |
| Primary site | Left lung | 37 (33.95%) | 67 (41.10%) | 0.724 (0.419, 1.241) | 0.246 |
|  | Right lung | 68 (62.39%) | 89 (54.60%) |  |  |
| Pathological type | Adenocarcinoma | 89 (81.65%) | 121 (74.23%) | 3.368 (1.191, 11.784) | 0.013 |
|  | Squamous cell carcinoma | 5 (6.58%) | 23 (14.11%) |  |  |
|  |  | Pleural**^+^** (n=107) | Pleural**^–^** (n=176) |  |  |
| Sex | Male | 46 (49.46%) | 83 (46.37%) | 1.131 (0.664, 11.784) | 0.701 |
|  | Female | 47 (47.71%) | 96 (53.63%) |  |  |
| Age | <60 | 31 (33.33%) | 66 (36.87%) | 0.857 (0.486, 1.495) | 0.596 |
|  | ≥60 | 62 (66.67%) | 113 (63.13%) |  |  |
| Smoking history | Yes | 25 (26.88%) | 83 (46.37%) | 0.442 (0.243, 0.784) | 0.004 |
|  | No | 65 (69.89%) | 95 (53.07%) |  |  |
| Primary site | Left lung | 32 (34.41%) | 72 (40.22%) | 0.802 (0.454, 1.404) | 0.426 |
|  | Right lung | 56 (60.22%) | 101 (56.42%) |  |  |
| Pathological type | Adenocarcinoma | 70 (75.27%) | 140 (78.21%) | 2.293 (0.806, 8.053) | 0.129 |
|  | Squamous cell carcinoma | 5 (5.38%) | 23 (12.85%) |  |  |
