# Supplementary figures and images for: Identification of nine mutant genes and establishment of three prediction models of organ tropism metastases of non‐small cell lung cancer
Source: Cancer Med. 2022 Sep 26;12(3):3089–100. doi: 10.1002/cam4.5233 (PMC9939125; doi:10.1002/cam4.5233)

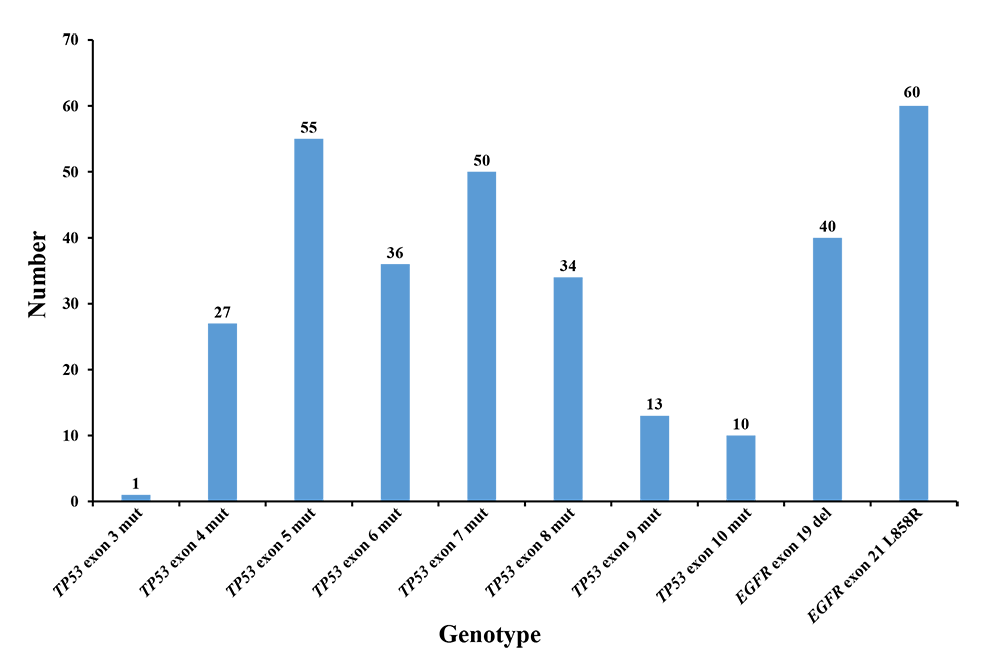

Supplement: Supplementary file 2 — Figure S1 [file CAM4-12-3089-s001.png]
